# Effective principal leadership behaviors to improve the teacher performance and the student achievement

**DOI:** 10.12688/f1000research.51549.2

**Published:** 2022-03-21

**Authors:** Jawatir Pardosi, Tria Ina Utari

**Affiliations:** 1 Faculty of Teacher Training and Education, University of Mulawarman, Samarinda, Kalimantan Timur, 75119, Indonesia; 2 Islamic education management, IAIN Ambon, Ambon, Maluku, 97128, Indonesia

**Keywords:** effective principal leadership behaviors, achievement

## Abstract

The principal’s role as a leader is crucial in improving the teacher performance and student achievement. However, it still remains unknown what kinds of school leadership behaviors that are effective to improve teacher performance and student achievement. This study aims to determine the level of significance of the effect of (1) the quality of principal's leadership behaviors on teacher performance, (2) the quality of principal's leadership behaviors on student achievement, and (3) the teacher performance on student achievement.This study is a quantitative descriptive research. The study population was 317 teachers with 281 teachers as the samples. The data collection technique was carried out through questionnaires, participant observation, and teacher performance appraisals. The data analysis technique used the Structural Equation Modeling (SEM). The results of this study showed a significant (5%) influence between: (1) the quality of the principal's leadership behaviors on the teacher performance where the estimated rate was 0.89 with the value of -t = 3.23> 1.96. The higher the quality of the principal's leadership behaviors had become, then it increased the quality of the teachers’ performance; (2) the quality of the principal's leadership behaviors on the level of student achievement where the estimated rate was 0.77 with the value of -t = 2.86> 1.96; and (3) the level of teacher performance on the level of learning achievement where the estimated rate was 0.92 with the value of -t = 4.45> 1.96. The structural model of learning outcomes showed that the loading factor of report cards had a greater effect of 0.995 when it compared to the school exam scores with an effect of 0.897. Effective principal leadership behaviors were limited to (a) the relationship between leaders and followers, (b) task structure, and (c) position power.

## Introduction

The leadership role of the principal is crucial to improve teachers’ performance. Consequently, high performance teachers will improve students’ learning outcomes. To improve teachers’ performance, a principal as the leader must have certain behaviors to accomodate the goal-oriented teaching-learning environment. Unfortunately, it remains unknown as to what kinds of successful and effective principal leadership behaviors will motivate teachers in improving their performance. Therefore, it is necessary to conduct a study to find the successful and effective leadership behaviors of school principals in accordance with the socio-cultural conditions of schools and teachers and other aspects that influence them.

Indonesia is a highly pluralistic country and diverse nation, reflected by its national credo: Bhinneka Tunggal Ika or Unity in Diversity. Mission of national education is conducted democratically, equally and non-discriminatorily based on human rights, religious values, cultural values, and national pluralism. Educational streaming consists of formal education, nonformal education, and informal education that may complement and enrich each other ( Act of the Indonesia, Number 20, Year 2003, Chapter VI, Article 13, Verse, 1).

School principals play an important role in improving education quality and accountability of education implementation in the educational unit. The problem commonly encountered from the principal is the weakness in managerial competence, while from the school supervisor is the lack of competence in supervisory skill.

In several past literature studies, there are differences in the formulation of the leadership variable, differences in the form of teacher performance as a moderate variable, and the achievement results used. This study looks at the effectiveness of leadership behavior generated through respondents' responses, in this case the school involved in the relationship between leaders and followers, the assignment structure, how with the principle of diversity in Indonesia, a leader is able to formulate goals, tasks, and solve plural problems. The position of the leader is also a concern of researchers. The principle of diversity that exists within the Unitary State of the Republic of Indonesia, becomes a challenge in leading the various ethnic and cultural characters that exist, thus creating an effective pattern of behavior in overcoming and solving problems that exist in its territory. The learning achievement used is based on changes in assessments in Indonesia, which were initially centralized, then given the freedom for schools to determine and provide their own assessments based on the situation of students at school.

The findings on successful and effective principal leadership behavior based on direct experience (empirical sophisticated) are beneficial for efforts to improve the quality of education. The application of the successful and effective leadership behaviors in a school can be seen as a reference or if possible, as a role model for other schools to conduct the same method. With such hope, it is possible to improve the quality of Indonesia’s education system following national and international standards.

### Research questions

1) Is there a significant influence of effective principal leadership behaviors on teacher performance?
2) Is there a significant influence of effective principal leadership behaviors on student achievement?
3) Is there a significant effect of teacher performance on student achievement?

## Literature review

### Conceptions of leadership and effective principal leadership behaviors

Formulating a definition of leadership that is acceptable to all groups and up to date is difficult because it is broad and complex and can be viewed from various aspects. Avolio (1981) has briefly reviewed some classifications of leadership: as a focus of group processes, as personality and its effects, as the inducing compliance, as the exercise of influence, as an act or behavior, as a form of persuasion, as a power relation, as an instrument of goal achievement, as an emerging effect of interaction, as a differentiated role, and as the initiation of structure.

Principal leadership is the principal's effort to influence, encourage, guide, and direct teachers, staff, students, parents, and other related individuals to work together in achieving set goals. To instill this role, the principal must show a persuasive and exemplary attitude. The principal as a leader has to realize that the success of the school life for which they are responsible is very much determined by their behavior.

The importance of leadership is in line with the opinion of Vroom, Yetton, and Fieler in Hendarman (2015) who believe that “leadership plays an important role in improving the performance of subordinates”. Smith in Hendarman (2015) argues that “to build an effective school there requires strong instructional leadership, clear attention to learning outcomes, high appreciation for students, a good environment and supervision at the level of achievement”. Assuming someone’s leadership is more effective, it will further improve the teacher performance and student achievement.

### Effective principal leadership behaviors

Based on the development of leadership theory, it is known that the trait theory views the effectiveness of leadership as largely determined by traits such as self-esteem, initiative, intelligence, language fluency, and creativity, including the physical characteristics possessed by a person as a manager.

Douglas (1967), the Blake and Houston Managerial Grid, the Ohio State Study and the Michigan study are developed by social psychologists, Rensis and Likert. This theory concerns the attention to two aspects of leadership behavior, namely the functions and leadership styles. In order for a group to run effectively, one must carry out two functions related to group relationships.

A situational approach (Contingency approach) depends on the situation, tasks, members, organization and other environmental variables. Well-known situational theories include Fiedler's contingency theory, Hersey and Blanchard's life cycle theory, and Sehmid and Tannembaun's series of leadership theory ( Fiedler & Chemers., 1974).

In its development, it is known that not every organization can use the same leadership approach. Some research indicates that a people-oriented leadership orientation tends to work more effectively. Meanwhile, several other studies have shown that a task-focused leadership orientation is more effective ( Feldmon & Arnold, 1983; Gorton & Schneider, 1991; Hoy & Miskel, 1987). The different results of leadership effectiveness occur due to the different organizational characteristics possessed by each group. The common question about the best leadership style does not actually refer to the best style, but it refers to the most effective style applied in a particular situation. The leader's behavior style can be an effective approach depending on the essential elements of the current situation of a group.

According to the Leadership Contingency Model developed by Fiedler (1967), there are three main situational variables to determine whether the situation is suitable or effective for the leadership: 1) the relationship between the leader and the members, 2) the degree of the task structure of the group (the relationship between the leader and the followers), and 3) the power and authority aspects where the position power is given power.

A principal sits in a strategic position in planning, implementing, and evaluating the school development programs because of the principal’s role as the highest leader in a school. Successful school principals can be seen based on their leadership style that is capable of influencing and mobilizing education stakeholders to jointly achieve school success with the same vision.

### Understanding teacher performance and student achievement

From various literature, it is known that the term ‘performance’ is used for the same purpose as the terms ‘work result’, ‘work performance’, and ‘performance’. Etymologically, performance comes from the word ‘to perform,’ which means to show or implement. The word performance means the act of performing; execution ( Webster, 2002).

Furthermore, Basrowi (2010) defines performance as the result or level of success of a person in the field of work according to certain criteria both in quality and quantity in carrying out their duties and responsibilities.

According to Woolf in Rusyan (2014), performance means the execution of an action. In other words, performance refers to the act of displaying or carrying out an activity. Therefore, it is no wonder for performance to often be defined as work appearance or work behavior. Performance is defined as the record of outcomes produced on specified job function or activity during a specified time period ( Russel, 1993). When it comes to teacher performance, it refers to the performance of teachers in planning, implementing and evaluating all learning activities.

The Georgia Department of Education has developed the teacher performance assessment instrument which has later been modified by the Indonesian Ministry of National Education to become the Teacher Ability Assessment Tools. The Teacher Ability Assessment Tools consist of teaching plans and materials or is known as the RPP (Learning Implementation Plan), classroom procedures, and interpersonal skills ( Priatna & Sukamto, 2013). Evaluation indicators for teacher performance are carried out on three learning activities in the classroom such as learning activity program planning, implementation of learning activities, and learning evaluation/assessment. Increasing the quality of teacher performance is considered as an effective policy in leading to substantial gains in student learning. From this perspective, the institution of teacher evaluation is a vital step in the drive to improve the effectiveness of teaching and learning as well as to level up the educational standards ( OECD, 2009).

## Methods

This study used a quantitative method. The quantitative method is carried out with a correlational survey to explain the relationship between variables after testing several hypotheses that have been formulated. The X variable represents the principal's effectiveness, the Y1 variable represents the teacher's performance, and Y2 is the student’s achievement which is divided into school exams and report card scores.

This research was conducted in 5 (five) State Senior High Schools (SMA) in Tenggarong, Kutai Kartanegara, East Kalimantan. These schools are SMA Negeri 1 and SMA Negeri 2 Tenggarong, SMA Negeri 3 Unggulan Tenggarong, and SMA Negeri 1 and SMA Negeri 2 Tenggarong Seberang. The population in this study amounted to 321 teachers. Determination of the sample using the Slovin formula with the desired level of precision is 98% or a significance of 0.02.

$$\text{Slovin}\;\text{formula}:\;n=\frac{321}{1+321{\left(0.02\right)}^{2}.}$$

From the above calculations, the total sample is 281 teachers. The questionnaires were distributed to 281 teachers of five different schools that made up the population of senior secondary schools in Tenggarong and at the same time those questionnaires were designed to pay more attention to the senior teachers who had long been titled as the civil servants in teaching senior high schools. Those five schools had many school achievements in the research object year of 2016-2018.

### Data collection

The primary data were collected through questionnaires. The questionnaire serves as a questionnaire to obtain primary data, namely data that comes from the first source. Then the data source in this study was the teachers who worked at the schools which were the research locations. This research instrument is arranged based on theoretical studies. Each variable of this study collects data related to exogenous variables, namely teacher performance (Y_1) and school achievement (Y_2) with endogenous variables, namely the effectiveness of the principal's leadership behavior pattern (X_1). All statements used to measure exogenous variables and endogenous variables were measured using a 5-level Likert scale, in which respondents were asked to state their demeanor by marking the statement very high until the gradation was very low. The data of student achievement were obtained from school documentation such as report cards and test scores (school exams).

Instrument testing was carried out on teachers who were taken from the population and were not sampled in the study. Testing or testing of validation instruments is carried out to what extent the accuracy and accuracy of a measuring instrument in performing its measuring function. Instrument validation was tested with the product moment formula (Pearson) using the SPSS version 20 application. The critical number limit is 0.05. The testing criteria is by comparing r _count_ with r _table_, if r _count_ > r _table_ then the instrument is considered valid, conversely if r _count_ < r _table_, it is considered invalid so it cannot be used in research. The calculation of reliability is to see the extent to which the measuring instrument can give relatively no different results when re-measuring the same symptoms at different times. So the measurement of reliability is related to the consistency and accuracy of the measurement. All valid instrument items were calculated for their reliability using Alpha Cronbach.

### Data analysis

The data on effectiveness of the principal's leadership behavior pattern on teacher performance and student achievement at the location were analyzed and tested using the multivariate Structural Equation Model (SEM) technique of the AMOS version 25 program.

Structural Equation Modeling (SEM) method as an analysis method that combines the factor analysis approach, structural modeling, and path analysis ( Sugiyono, 2017). This analysis method is also stated by Bagozzi and Fornell in Ghozali (2005) as a second generation multivariate analysis technique.

**Figure 1.**
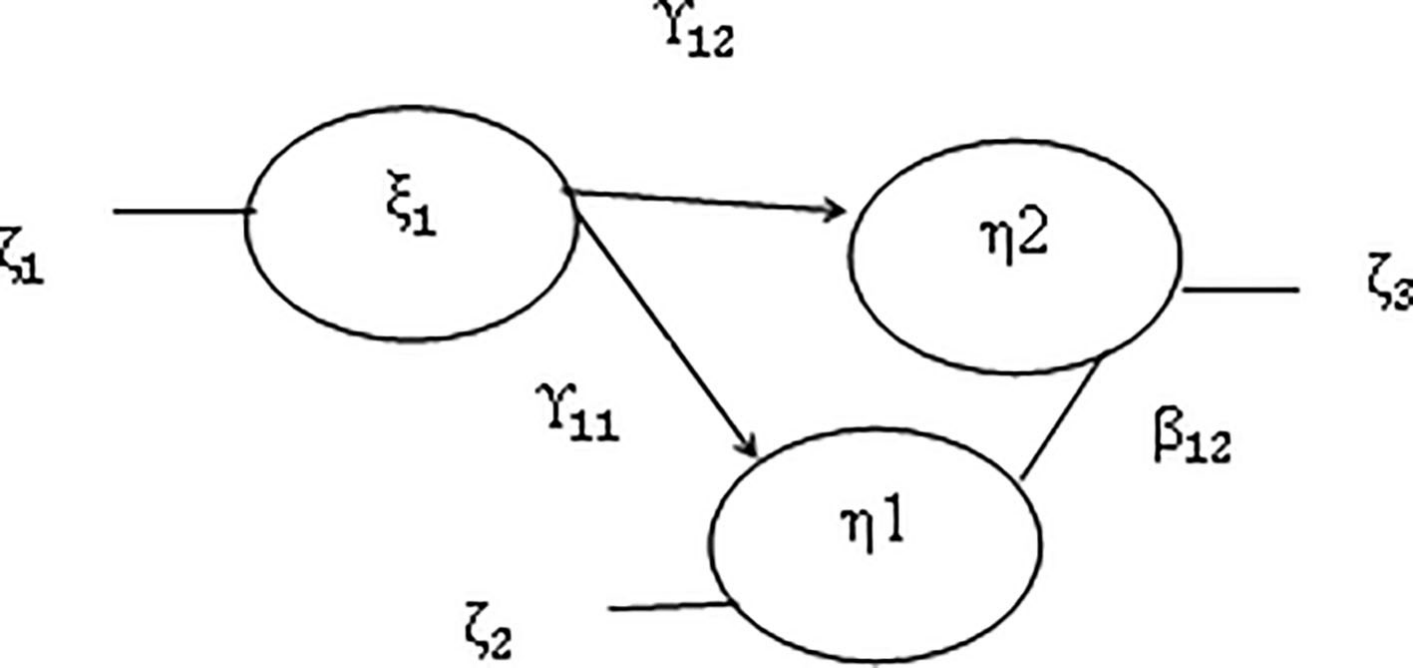
Structural model of the relationship between variables ( Sugiyono, 2017). Description: ξ_1 = latent variable effectiveness of leadership behavior patterns η1 = latent variable of teacher performance η2 = latent variable of student achievement γ_12 = coefficient of direct influence of variable effectiveness of leadership behavior patterns on student achievement. γ_11 = coefficient of direct influence of variable effectiveness of leadership behavior patterns on teacher performance. β_12 = coefficient of influence of teacher performance variables on student achievement variables ζ_1 = Measurement error effectiveness of leadership behavior patterns ζ_2 = Measurement error of teacher performance ζ_3 = Measurement error of student achievement.

• The measurement model is the relationship (loading value) between indicators and constructs (latent variables). The measurement model is also known as Confirmatory Factor Analysis (CFA). This phase aims to test the feasibility of constructing validity and indicator reliability. The measurement model is used to determine the validity of the manifest/measured variable, whether it can be used as an indicator of the latent variable. To see the size of the validity coefficient, it can be seen the size of the factor load (λ). The more the factor price (λ), the more valid the indicator will be. The measure to determine the value of the factor (λ) is said to be valid and can use the t value test (t-value). As Carmines and Zeller in Sugiyono (2017) explain that a good construct has a load factor of (λ) ≥ 0.30. To see the magnitude of the indicator reliability coefficient can see the value (1-δ) for exogenous variables and value (1-ε) for endogenous variables. The more the value (1-δ) and (1-ε), the more reliable the indicator is. Factor analysis in CFA is different from the factor analysis used in statistical/multivariate analysis. In Factor analysis, the CFA model is formed first and the number of latent variables is determined by the analysis. Then each construct has its indicators defined in advance based on the theory used. Meanwhile, statistical or multivariate factor analysis is more of an exploratory factor analysis. Factor content or factor loadings that connect the latent variable and the observed variable are given the notation λ (lambda), where on the x side is (Lambda x) and the y side is (Lambda y)
• The structural model is the relationship between independent and dependent constructs. Bollen (1989) in Ghozali (2012) combines testing of measurement and structural models that allows the researchers to test measurement errors as an integral part of SEM. In this phase, every construct and indicator that has been tested for validity and reliability will be reprocessed at the measurement model phase. This phase aims to estimate the structural model simultaneously, so that the relationship between the independent and dependent variables will be seen, as well as the measurement quality of the factor load value of each construct and indicator. Parameters indicating regression of endogenous latent variables on exogenous latent variables are labeled with the Greek letter γ (gamma). Parameters indicating regression of endogenous latent variables on other endogenous latent variables are labeled with the Greek letter β (beta). Structural model testing can be seen at the level (goodness of fit statistic) – see Figure 1.

The staps of the SEM procedure in general for Confirmatory Factor Analysis (CFA) in this study are as follows:

• Model specification
  The first step is to specify the research model being analyzed. Outline model specifications are as follows:
  1. Defining latent variables, namely the effectiveness of leadership behavior patterns, teacher performance and student achievement.
  2. Defining the unobserved variables, namely the relationship between leaders and followers; follower belief, respect, familiarity, compassion for the leader. Quality of task structure relationships; clarity of objectives, clarity of tasks to be carried out, diversity of problem solutions faced, openness of the correctness of decisions to other parties regarding specific decisions faced in the work, the number of correct problem solutions to the specifics of decisions. Quality of standing strength; power directing followers, evaluating followers, authority giving reward and punish to followers. The ability to formulate learning device plans, the ability to plan learning activities, the ability to choose learning sources and media, the ability to encourage and maintain involvement, the ability to apply learning approaches and strategies, the ability to end effective learning, the ability to design assessment methods and evaluation tools. School exam scores and report cards.
• Quantitative data collection is carried out through surveys (primary data) in accordance with the instrument questionnaire created.
• Operating the SIMPLIS program and analyze its output. SIMPLIS program which is the result of respecification.
  Broadly speaking, the analysis of the output of the SIMPLIS program is as follows:
  1. Checking for the offending estimate, such as negative error variance and standardized loading factor, which most often occur is greater than 1.0, and the standard error value is very large.
  2. Checking the validity of the observed variables, the criterion for good validity is if the value of standardized factor loadings is ≥ 0.30.
  3. Analyzing the reliability of the measurement model, looking at the value (1-δ) for the exogenous variable and the value (1-ε) for the endogenous variable. The greater the value (1-δ) and (1-ε), the more reliable the indicator is.
  4. Testing the suitability or Goodness of Fit Statistic (GOF).
• Respecification of the research model and changes to the SIMPLIS program. Respecification is carried out when there are offending estimates, the validity of the model is not good enough, the overall fit and the reliability of the model is not good enough. To carry out model respecification, we make changes to the SIMPLIS program according to the requirements of the specification.

### Ethical approval

Ethics approval was obtained from schools in Kutai Kartanegara based on the application for research permits letter number 050/UN17.5.35.7/PG/2018. In filling out the questionnaire the teacher is asked to fill in anonymously, and is informed in advance that the statement given is privacy and will not affect any assessment and justification.

## Results

The results of the descriptive statistical measurement of the effectiveness of the principal's leadership behavior, the average score (mean) obtained is 108.85. Looking at the standard deviation of data processing using SPSS or standard deviation of 22,959 with a median value of 122.00 and a value that often appears (mode) of 132.23. Teacher performance variable, has an average score (mean) is 115.26. Looking at the acquisition of the standard deviation or standard deviation of 19,975 with a median value of 122.00 and a value that occurs frequently (mode) of 133.80. The student achievement variable, the average score (mean) obtained for the US score is 80.74 and the report card score is 81.80. Looking at the standard deviation of the US value of 5,742 and 5,024 on the report card value, with a median value of 82.00 on the US value and 84.00 on the report card value, as well as the frequently occurring value (mode) of 86 on the US value, and 86.75. Furthermore, it is included in the evaluation of research models in structural analysis of equation models. SEM analysis requires data to be normally distributed to avoid bias in data interpretation that can affect other data. Data normality measurement is carried out simultaneously with the model suitability test process.

### Checking data outliers

The results of the calculations in Table 1 showed the existence of outliers where some data cleaning was required. Any data containing the probability p1 or p2 with a value of less than 0.05 was proven to experience outliers. Data that were free from outliers must have the value of p1 or p2 > 0.05 to make sure that there was no difference of significance between data and data groups.

**Table 1.** Observations farthest from the centroid (Mahalanobis distance).

Observation number	Mahalanobis d-squared	p1	p2
93	78.005	.083	1.000
44	77.823	.085	.998
60	77.206	.092	.995
69	76.827	.097	.988
83	76.467	.102	.975
3	76.123	.107	.955
62	75.351	.119	.951
.	.	.	.

### Checking data normality

Based on the normality test, the results obtained were presented in Table 2.

**Table 2.** Assessment of normality after data outlier cleaning had been done.

Var	Min	Max	Skew	Kurtosis	c.r
Y2.2	70.000	90.000	−.679	−.244	−.491
Y2.1	70.000	90.000	−.331	−.876	−1.761
Y1.7.1	2.000	5.000	−.378	−1.043	−2.096
Y1.7.2	2.000	5.000	−.214	−.919	−1.849
.	.	.	.	.	.
Multivariate				20.717	1.145

In Table 2, the data was normal with a value of c.r (critical ratio) 1.145 fulfilled the requirements for normality data, namely −2.58 <c.r <2.58. There was no more data to discard because all data were multivariate normal. This study consisted of one exogenous variable known as the Effectiveness of Leadership Behavior Patterns and two endogenous variables known as the Teacher Performance and Student Achievement, which were referred to as latent variables.

After the data is declared normal, the suitability of the model will be tested by looking at the Goodness of Fit value with the criteria being the reference value being Chi-Square, Probabilitas, Root Mean Square of Approximation (RMSEA), Adjusted Goodness of Fit Index (AGFI), Tucker-Lewis Index (TLI), Comparative Fit Index (CFI), Normed Fit Index (NFI), and ChiSquare X ^2^ relative/degree of freedom (CMIN/DF) produced.

### Goodness of Fit Statistic (GOF)

Evaluation of the proposed research model through Goodness of Fit analysis can be seen in Table 3.

**Table 3.** Goodness of fit.

No	Statistics	Criteria Fit	Result	Description
1	X ^2^	P > 0.05	2851.3	Fit
2	Sig Prob.	≥0.90	.781	Fit
3	RMSEA	≤ 0.08	.0694	Fit Marginal
4	AGFI	≥0.90	.981	Fit
5	TLI	≥0.90	.975	Fit
6	CFI	≥0.90	.950	Fit
7	NFI	≥0.90	.962	Fit
8	CMIN/DF	≤2.0	1.764	Fit

The purpose of chi-square analysis is to develop and to test a model that fits the data. A high chi-square value will result in a probability value (p) that is smaller than the significance level (α). This shows that the input covariance matrix between predictions and actual observations is not significantly different ( Ghozali, 2012).

This test refers to a high value and a significance level less than 0.05, which indicates that there is no significant difference between the estimated covariance matrices. Chi square is very sensitive to sample size. After the initial model was processed with a sample size of 281 respondents, the value in this study was 2851.3 with a probability of 0.781. This shows that the proposed research model is acceptable because there are differences between the sample covariance matrices with the observed population covariance matrices. The RMSEA value of 0.0694 is smaller than 0.08. The AGFI value of 0.981 is close to 1. The TLI value of 0.975 is close to 1. The CFI value of 0.950 is close to 1. The NFI value of 0.962 is close to 1. The CMIN/DF value of 1.764 is smaller than 2.0.

Based on the measurement of the overall goodness of fit research model (after the modification process had been conducted), it showed that the model proposed in this study was well accepted.

### The identification of the structural model

The identification of the structural model could be seen from the results of variables calculation by calculating the amount of covariance data and the variance compared to the number of parameters to be estimated. The model output was presented in the following Table 4.

**Table 4.** Estimasi parameter covarian data and the variance.

Number of distinct sample moments:	1830
Number of distinct parameters to be estimated:	126
Degrees of freedom (1830-126):	1704

Result (default model)

Minimum achieved

Chi-square = 2851.3

Degrees of freedom = 1704

Probability level = .781

Based on the notes for the above model, the result showed the number of samples was N = 281, the total number of covariance data was 1,830 while the number of parameters to be estimated was 126. The resulting degree of freedom was 1.704, because 1.704> 0 (positive df) as stated by the sentence “minimum achieved”. The process of estimating the probability maximum had been performed and the estimation was identified by the resulting data with a normal distribution.

### Testing of Structural model

Structural model testing is done by analyzing the significance level of the causality relationship between constructs in the model which is based on the estimated value of the structural coefficient with the t value of each parameter. It is known that the t-table value with a significance level of 5% = 1.96. Analysis of the relationships between constructs is shown by the regression weight value.

There was a direct and significant influence between the effectiveness of principal leadership behaviors on teacher performance. Based on the test results in Table 5 the estimated rate was 0.89 with the value -t = 3.23 > 1.96. This indicated a significant and positive influence between the effectiveness of leadership behaviors on teacher performance. Hence, the H1 showed a direct influence between the effectiveness of leadership behavior patterns on the teacher performance supported at the 5% significance level. The results above explain that the higher the level of principal leadership behaviors effectiveness was, the higher rate of teacher performance effectiveness would be.

**Table 5.** Regression weight.

Regression weight			Estimate	S.E	C.R	P
Teacher Performance	←	Effectiveness of Leadership Behavior Patterns	.894	3.23	6.024	***
Student Achievement	←	Effectiveness of Leadership Behavior Patterns	.769	2.86	4.110	***
Student Achievement	←	Teacher Performance	.923	4.45	5.964	***

There was a significant direct effect between the effectiveness of the principal's leadership behavior on the student learning achievement. Based on the test results in Table 5 the estimated rate was 0.77 with the value -t = 2.86 > 1.96 showed a significant and positive influence between the effectiveness of leadership behavior patterns on the student learning achievement. Hence, H1 indicated a direct influence between the effectiveness of leadership behavior patterns on the student achievement supported at the 5% of the significance level. This result explained that the higher the level of effectiveness of the principal's leadership behavior, the higher the student learning achievement would become.

There was a direct influence between teacher performance on student learning achievement. Based on the test results in Table 5 the estimated rate was 0.92 with the value -t = 4.45 > 1.96 which indicated a significant and positive influence between the teacher performance and the student learning achievement. The direct effect of teacher performance on student achievement was 5% of the significance level. This result showed that the higher the teacher performance became, the more the student achievement could be improved.

***a) Effective principal leadership behaviors***

Based on the results of quantitative analysis, the variable of effective of high school leadership behaviors in the measurement model of leadership behaviors showed that the relationship between leaders and followers (X1) had the greatest influence in the contribution of latent variables to the effective principal leadership behavior 0.72, task structure (X2) 0,69, and the position leader (X3) 0.57.Koefisien reliabilitas dari the relationship between leaders and followers (X1) 0.39, task structure (X2) 0, 42, and the position leader (X3) 0.21.

The results of this study indicate the principal leadership behaviors that had been analyzed from the dimensions of leadership and follower relations, task structure, and leadership positions was highly effective. The essence of the principal's existence as a key person in the organization must be improved. Interested in taking various actions to achieve the vision, mission and goals of the organization. A leader is a vision to provide directions in which the organization will be taken. The principal as a management duty bearer must continue to refer to the organization's visions and present himself as a visionary role model.

Based on the above results, it could be concluded that the relationship factor between leaders and followers, task structure, and leader position possessed a significant effect (contributes) in constructing the effectiveness of principal leadership behaviorsin schools.

***b) Teacher performance variables***

Based on quantitative analysis, the measurement of teacher performance variables showed the level of ability to load the formulation of the RPP factor (Y_1) had an effect of 0.68, the level of ability to plan learning activities (Y_2) had an effect of 0.57, the level of ability to choose sources and learning media (Y_3) had an effect effect of 0.58, the level of ability to apply learning strategies and approaches (Y_4) had an effect of 0.50, the level of ability to encourage and maintain student involvement (Y_5) had an effect of 0.46, the level of ability to end learning effectively (Y_6) had an effect of 0.73, and the level of ability to design assessment methods and evaluation tools (Y_7) had the greatest effect of 0.57. Koefisien reliabilitas dari the level of ability to load the formulation of the RPP factor (Y_1) had an effect of 0.51, the level of ability to plan learning activities (Y_2) had an effect of 0.55, the level of ability to choose sources and learning media (Y_3) had an effect effect of 0.53, the level of ability to apply learning strategies and approaches (Y_4) had an effect of 0.46, the level of ability to encourage and maintain student involvement (Y_5) had an effect of 0.48, the level of ability to end learning effectively (Y_6) had an effect of 0.43, and the level of ability to design assessment methods and evaluation tools (Y_7) had the greatest effect of 0.55.

**Figure 2.**
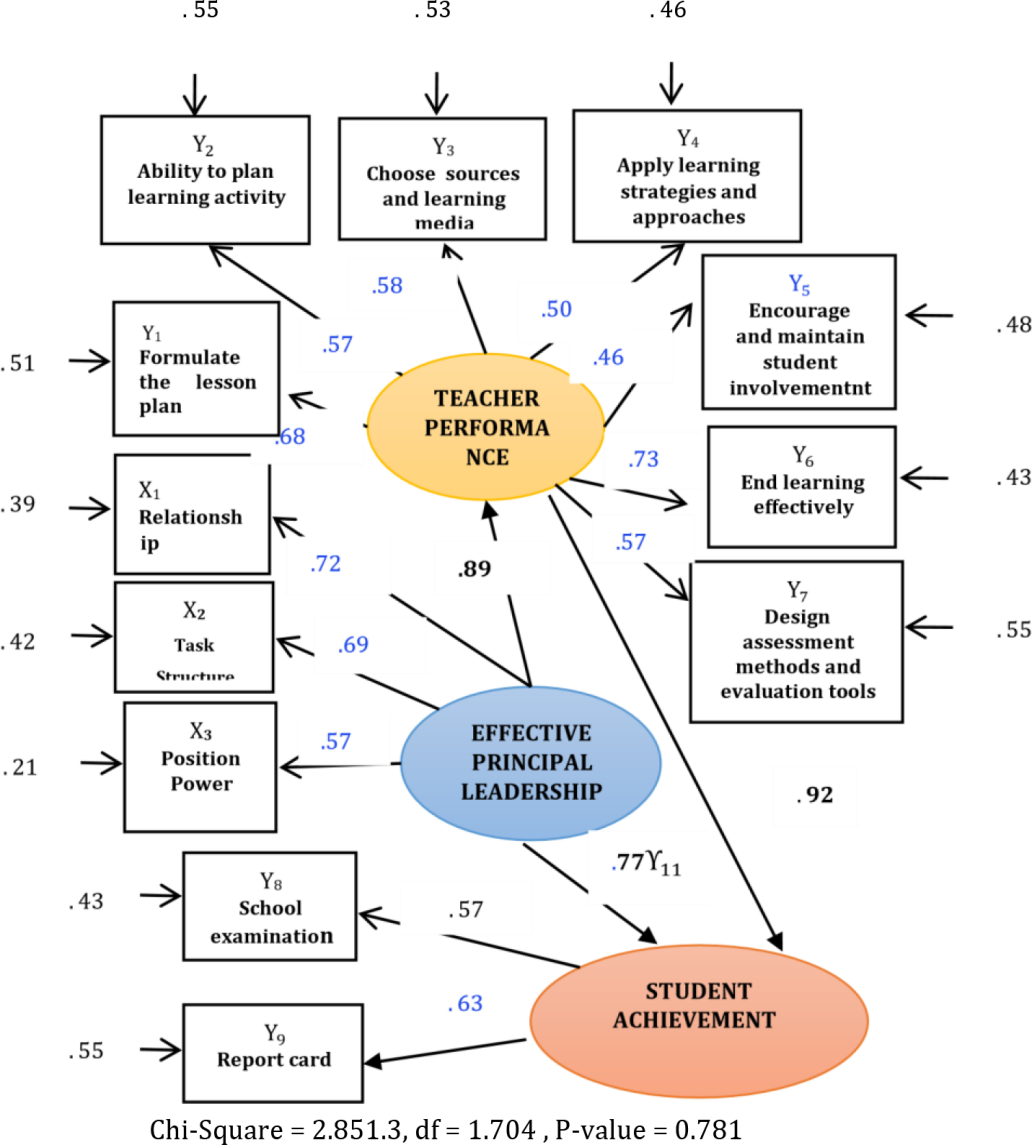
The influence model of effective principal leadership behaviors on teacherperformance and student achievement. Note: Value of Standardized loading factor 0.30 Carmines and Zeller in Sugiyono (2017).

Based on Figure 2, it could be concluded that the level of ability of the factors to formulate lesson plans (Y1), the level of ability to plan learning activities (Y2), the level of ability to choose resources and learning media (Y3), the level of ability to apply learning approaches and strategies (Y4), the ability to encourage and maintain student involvement (Y5), the ability to end learning effectively (Y6), and the ability to design assessment methods and evaluation tools (Y7) possessed a significant effect on teacher performance.

***c) Student achievement***

The model of measuring student achievement showed that the school examination loading factor had an effect of 0.57 and the report card (NR) had an effect of 0.63.Koefisien reliabilitas dari that the school examination loading factor had an effect of 0.43 and the report card (NR) had an effect of 0.55. Based on the data, the school exam factors and report card scores possessed a significant effect (contribution) on the construction of student achievement in high schools.

## Discussion

In conducting this analysis, bias is prone to occur so that it is necessary to fulfill the requirements. Crude data in the form of question instruments was tested first for validation. The instrument data in the form of validation test questionnaires were distributed as many as 40 questionnaires and only 30 validation test questionnaires were returned. The crude data of the validity test regarding the variable effectiveness of the Principal's Leadership Behavior Pattern contained an instrument of 40 statement items. The teacher performance variable contains an instrument of 40 statement items. Meanwhile, the student learning achievement variable data was obtained from the documentation in the form of archives of school examination scores and report card scores.

Based on significance; If the significance value> 0.05 then the item is declared invalid, but if the significance value < 0.05 then the item is declared valid. Then the instrument data that is declared valid are; this is a valid instrument for 30 items and 10 items invalid on the variable of the Effectiveness of Leadership Behavior Patterns. There are 30 valid instruments for teacher performance and 10 invalid items. The method of decision making for reliability testing uses a limit of 0.6. Reliability is less than 0.6, it is not good, while 0.7 is acceptable, and 0.8 is good. From the results of the calculation of the reliability value of Cronbach's Alfa for the variable effectiveness of leadership behavior patterns of 0.978, it can be concluded that it is declared reliable, with good reliability. For the teacher performance variable in the reliability table of 0.975, it can be declared reliable, with good reliability.

The Variable Effectiveness of Leadership Behavior Patterns was compiled with a questionnaire of three indicators, which were divided into 30 questions with the highest score of 5 and the lowest score 1. The Effectiveness of Leadership Behavior Patterns in SMA Negeri Tenggarong had the highest score of 148, and the lowest score was 46. With a value range of 102 The average score (mean) obtained by 281 respondents was 108.85. The acquisition of standard deviation from data processing or standard deviation is 22,959 with a median value of 122.00 and a frequently occurring value (mode) of 132.23.

The teacher performance variables were arranged in a questionnaire with seven indicators. The performance of public high school teachers in Tenggarong has the highest score of 150, and the lowest score of 56. With a range of scores of 94, the average score (mean) obtained by 281 respondents was 115.26. The acquisition of standard deviation from data processing or standard deviation is 19,975 with a median value of 122.00 and a frequently occurring value (mode) of 133.80.

Student achievement variables are arranged based on documentation data obtained from the school. Based on the data collected, it is obtained data about the achievement of state high school students in Tenggarong, namely data on school examination scores and report cards with the highest score of 90, and the lowest score of 70, with a value range of 20. The average score (mean) obtained by 281 respondents on the school exam score was 80.74 and the report card score was 81.80. The acquisition of standard deviation from data processing or standard deviation on school exam scores of 5,742 and 5,024 on report cards. Based on the questionnaire that has been given to 281 respondents, the total sample size of 281 respondents is feasible to be processed and according to the required requirements where the Structural Equation Modeling (SEM) technique requires 100-200 samples.

Evaluation of the proposed research model through Goodness of Fit analysis. The value in this study is 2851.3 with a probability of 0.781 which indicates that the proposed research model is acceptable because there is a difference between the sample covariance matrices and the observed population covariance matrices. The Goodness Of Fit Index (GFI) reflects the overall level of suitability of the model which is calculated from the squared residuals of the predicted model compared to the actual data. Recommended acceptance rate value is ≥ 0.90. RMSEA is a measure that tries to improve the tendency of the chi square statistic to reject models with large sample sizes. Recommended acceptance rate value is ≤ 0.08. AGFI is a development of the Goodness Of Fit Index (GFI) which has been adjusted to the ratio between the proposed degree of freedom model and the degree of freedom from null model. AGFI's recommended acceptance rate value is ≥ 0.90. TLI as a tool for evaluating factor analysis combines the parsimony size into the index of comparison between the proposed model and the null model. Recommended acceptance rate value is ≥ 0.90. CFI is an incremental fit index that compares the tested model with the null model. The recommended value is ≥ 0.90. NFI is a measure of comparison between the proposed model and the null model. Recommended acceptance rate value is ≥ 0.90. CMIN/DF is a measure obtained from the chi square value divided by the degree of freedom. This index is a parsimonious suitability index that regulates the relationship between the goodness of fit of the model and the number of estimated coefficients expected to achieve the level of conformity. The recommended value is ≤ 2.0.

The variable effectiveness of leadership behavior patterns on teacher performance and student achievement in SMA Negeri Tenggarong shows a significant influence between the effectiveness of leadership behavior patterns on teacher performance of 0.89. The effectiveness of leadership behavior patterns on student achievement was 0.77. And teacher performance on student achievement has a higher direct effect of 0.92. Analysis of the effectiveness assessment of the principal's behavior patterns in terms of the relationship to followers, task structure, and position showed a high enough level of approval at 71.17%. This means that what the principal has done for the variable effectiveness of leadership behavior patterns is good enough. So this explains that the respondents quite agree with the statements given in the questionnaire. However, 13.53% of respondents stated that the category was low. This needs to be an evaluation for the principal. Apart from having the ability to influence others, what is more important for the principal is the ability to inspire other parties. So that they are proactively motivated to take various actions in order to achieve the vision, mission and goals of the organization.

Teacher performance obtained a fairly high response of 65.13% of respondents agreed with what was the question in the questionnaire, namely the ability to formulate lesson plans, the ability to plan learning activities, the ability to choose learning sources and media, the ability to encourage and maintain involvement, the ability to apply learning approaches and strategies, the ability to end Effective Learning, Ability to Design Assessment Methods and Evaluation Tools. Through the ability to formulate a good lesson plan, a teacher is easier and more focused in carrying out learning activities which in turn will improve student achievement. However, 13.88% of respondents stated that it was quite low.

School is an institution specially designed for teaching students under the supervision of teachers. The principal can increase the stimulus in relation to teacher performance. So that the higher the effectiveness of the principal's leadership behavior pattern, the higher the teacher's performance is. The response to business can be successful or unsuccessful. Since the principal's primary responsibility in the organization is to carry out work with and through people, his success is measured by the output or productivity of the group he leads. Good teacher performance can increase productivity in class in learning so that it can produce high student learning achievement. Likewise, on the other hand, poor performance can have an impact on student achievement in the research location.

When analyzing the effectiveness of the principal's leadership behavior pattern, the measurement results show that the loading of the leader-follower relationship factor has the greatest influence in the contribution of the variable to the effectiveness of the leadership behavior pattern. The model for measuring the effectiveness of leadership behavior patterns can be used as an illustration that the principal needs to pay attention to the aspect of the position of the leader that has the smallest contribution. As for the level of the leader's strength in directing, evaluating, the authority of the leader in giving rewards, giving punishment and problem-solving abilities as well as the level of openness and diversity in solving problems for his followers.

The results of this study conclude that there is a positive relationship between the effectiveness of leadership behavior patterns on teacher performance and student achievement in 5 (five) State Senior High Schools in Tenggarong, Kutai Kartanegara, East Kalimantan. However, the authors recognize and acknowledge that this study has weaknesses and limitations, both limited knowledge and planning when carrying out research in the field. This study only examines three factors that are thought to influence the effectiveness of leadership behavior patterns, namely teacher performance and student achievement. This is done because of the limitations of the author in terms of time, cost, and energy, so the researchers hope that further research can be developed on other factors.

The implication of this research is that the relationship between leaders and followers in the behavioral effectiveness of leadership has the highest contribution value, so that if this good relationship can continue, it is believed to be able to improve student achievement and teacher performance. However, in the results obtained, schools need to be concerned about teacher performance, 13.88% of respondents stated that it was quite low. This can be seen from the results of statistical measurements of teacher respondents' answers, the level of ability to encourage and maintain student involvement has the least influence and the level of ability to apply learning approaches and strategies. So if schools want to develop, they need to increase/focus on evaluating and developing learning approaches and strategies by teachers, as well as increasing student involvement in classroom learning.

## Conclusions

The relationship between the leader and the followers, the task structure, and the position of the leader give a significant influence (contribution) to the construct of the effectiveness of leadership behavior patterns in 5 (five) Senior High Schools in Tenggarong. This shows the existence of the principal as a leader with high effectiveness. Leaders must seek to influence followers in various ways, such as using legitimate authority, creating models (being role models), setting goals, rewarding and punishing, organizational restructuring, and communicating a vision even though different results can be obtained from different organizations. Thus, this research can be viewed as a detailed description of what will be carried out, namely that a leader can be considered effective if he can persuade his followers to leave their personal interests for organizational success in the necessary situations. The factor of the level of ability to formulate learning device plans, the level of ability to plan learning activities, the level of ability to choose learning sources and media, the level of ability to apply learning approaches and strategies, the level of ability to encourage and maintain student involvement, the level of ability to end learning effectively, and the level of ability to design methods assessment and evaluation tools provide a significant influence (contribution) to the construct of teacher performance in 5 (five) Senior High Schools (SMA) in Tenggarong, Kutai Kartanegara, East Kalimantan. The form of teacher performance carried out in the learning process activities in this study is assumed to be an assessing factor for becoming a future teacher.

## Data availability

### Underlying data

Figshare. Effective principal leadership behaviors to improve teacher performance and student achievement. Dataset. https://doi.org/10.6084/m9.figshare.14398577.v4 ( Utari et al., 2021).

This project contains the following underlying data:

- This is the result of a questionnaire recapitulation of 281 teacher respondents.

Data are available under the terms of the Creative Commons Attribution 4.0 International license (CC-BY 4.0).
