# Supplementary material for: Effective principal leadership behaviors to improve the teacher performance and the student achievement
Source: F1000Res. 2022 Mar 21;10:465. Originally published 2021 Jun 14. [Version 2] doi: 10.12688/f1000research.51549.2 (PMC9168782; doi:10.12688/f1000research.51549.2)
Supplement: Peer review history

# Peer review: Reviewer response for version 2

**DOI:** 10.5256/f1000research.122149.r128175

**Published:** 2022-05-27

**Authors:** Lokman Mohd Tahir

**Affiliations:** 1 School of Education, Universiti Teknologi Malaysia, Skudai, Malaysia

I have looked at the recent revision. It is considered as accepted since the authors have made some improvements to the content of the manuscript.

Is the work clearly and accurately presented and does it cite the current literature?

Partly

If applicable, is the statistical analysis and its interpretation appropriate?

Yes

Are all the source data underlying the results available to ensure full reproducibility?

Yes

Is the study design appropriate and is the work technically sound?

Yes

Are the conclusions drawn adequately supported by the results?

Yes

Are sufficient details of methods and analysis provided to allow replication by others?

Partly

Reviewer Expertise:

NA

I confirm that I have read this submission and believe that I have an appropriate level of expertise to confirm that it is of an acceptable scientific standard.

# Peer review: Reviewer response for version 2

**DOI:** 10.5256/f1000research.122149.r128176

**Published:** 2022-04-14

**Authors:** Huang Wu

**Affiliations:** 1 Department of Educational Leadership, Research and Technology, Western Michigan University, Kalamazoo, MI, USA

The authors have addressed all issues I raised.

Is the work clearly and accurately presented and does it cite the current literature?

Partly

If applicable, is the statistical analysis and its interpretation appropriate?

Partly

Are all the source data underlying the results available to ensure full reproducibility?

Partly

Is the study design appropriate and is the work technically sound?

Partly

Are the conclusions drawn adequately supported by the results?

Partly

Are sufficient details of methods and analysis provided to allow replication by others?

Partly

Reviewer Expertise:

Educational Leadership

I confirm that I have read this submission and believe that I have an appropriate level of expertise to confirm that it is of an acceptable scientific standard.

# Peer review: Reviewer response for version 1

**DOI:** 10.5256/f1000research.54731.r98983

**Published:** 2022-01-11

**Authors:** Huang Wu

**Affiliations:** 1 Department of Educational Leadership, Research and Technology, Western Michigan University, Kalamazoo, MI, USA

Thanks for the invitation to review this study. I have enjoyed reading this study which addresses an important topic in the context of Indonesia. I hope my suggestions are helpful in revising and improving this manuscript.

 First, the underlying rationale for the study needs further development. Several questions should be addressed when revising the introduction. There are numerous studies that have examined the relationship between principal leadership and student achievement (e.g., Liebowitz & Porter, 2019 ^1^; Wu *et al*., 2020a ^2^, 2020b ^3^; Wu & Shen, 2022 ^4^; Shen *et al*., 2021 ^5^; Heck & Hallinger, 2009 ^6^; Heck & Hallinger, 2010 ^7^). What are the deficiencies in past literature? What gap in the literature did the authors identify?

 In the introduction, the authors argued that “ *it remains unknown as to what kinds of successful and effective principal leadership behaviors will motivate teachers in improving their performance.*” However, this study did not address this question. In this study, the research questions the authors addressed are “whether principal leadership impacts student achievement and teacher performance” rather than “what kinds of leadership behaviors will impact student achievement and teacher performance”.

 Second, I think a detailed review of previous studies that examined the relationship between principal leadership and student achievement/teacher performance is needed. Also, this study was conducted in Indonesia. Is it possible for the authors to briefly introduce to readers principal leadership in Indonesia? Principal leadership effects were well examined in a western context. The Indonesian context would be one contribution of this study.

 Third, there are some comments about your data analysis procedure and results:

- Could you please provide the readers with more details about how principal leadership, teacher performance, and student achievement were measured?
- I think the section of the SEM introduction could be simplified (page 5). Figure 1 is problematic.
- The arrow from η1 to η2 is missing.
- What does C.R. mean in Table 5? Confidence interval? Please report the exact p-value rather than asterisks.
- Could you please provide descriptive statistics?
- Can you calculate the indirect effect of principal leadership on student achievement through teacher performance?

Fourth, the discussion section should discuss the implications of this study for research, policy, and practice. Currently, the discussion section reads like results reporting. Also, please discuss your findings in a more organized way. I would suggest the authors discuss this study’s implication for future research, policy, and practice, respectively in the context of Indonesia.

Is the work clearly and accurately presented and does it cite the current literature?

Partly

If applicable, is the statistical analysis and its interpretation appropriate?

Partly

Are all the source data underlying the results available to ensure full reproducibility?

Partly

Is the study design appropriate and is the work technically sound?

Partly

Are the conclusions drawn adequately supported by the results?

Partly

Are sufficient details of methods and analysis provided to allow replication by others?

Partly

Reviewer Expertise:

Educational Leadership

I confirm that I have read this submission and believe that I have an appropriate level of expertise to confirm that it is of an acceptable scientific standard, however I have significant reservations, as outlined above.

## References

[1] : The Effect of Principal Behaviors on Student, Teacher, and School Outcomes: A Systematic Review and Meta-Analysis of the Empirical Literature. *Review of Educational Research* .2019;89(5) : 10.3102/0034654319866133 785-827 10.3102/0034654319866133

[2] : Principal leadership effects on student achievement: a multilevel analysis using Programme for International Student Assessment 2015 data. *Educational Studies* .2020;46(3) : 10.1080/03055698.2019.1584853 316-336 10.1080/03055698.2019.1584853

[3] : Examining the effect of principal leadership on student science achievement. *International Journal of Science Education* .2020;42(6) : 10.1080/09500693.2020.1747664 1017-1039 10.1080/09500693.2020.1747664

[4] : The association between principal leadership and student achievement: A multivariate meta-meta-analysis. *Educational Research Review* .2022;35: 10.1016/j.edurev.2021.100423 10.1016/j.edurev.2021.100423

[5] : The relationship between growth in principal leadership and growth in school performance: The teacher perspective. *Studies in Educational Evaluation* .2021;70: 10.1016/j.stueduc.2021.101023 10.1016/j.stueduc.2021.101023

[6] : Assessing the Contribution of Distributed Leadership to School Improvement and Growth in Math Achievement. *American Educational Research Journal* .2009;46(3) : 10.3102/0002831209340042 659-689 10.3102/0002831209340042

[7] : Testing a longitudinal model of distributed leadership effects on school improvement. *The Leadership Quarterly* .2010;21(5) : 10.1016/j.leaqua.2010.07.013 867-885 10.1016/j.leaqua.2010.07.013

# Response

**Published:** 2022-01-31

**Authors:** tria ina utari

**Affiliations:** IAIN AMBON, Indonesia

Thank you for taking the time and revising quickly to improve this manuscript.

 In several past literature studies, there are differences in the formulation of the leadership variable, differences in the form of teacher performance as a moderate variable, and the achievement results used. This study looks at the effectiveness of leadership behavior generated through respondents' responses. In this case, the school is involved in the relationship between leaders and followers, the assignment structure, how with the principle of diversity in Indonesia, a leader is able to formulate goals, tasks, and solve plural problems. The position of the leader is also a concern of researchers. The principle of diversity that exists within the Unitary State of the Republic of Indonesia becomes a challenge in leading the various ethnic and cultural characters that exist, thus giving rise to an effective pattern of behavior in overcoming and solving problems that exist in its territory. The learning achievement used is based on changes in assessments in Indonesia, which were initially centralized, then given the freedom for schools to determine and provide their own assessments based on the situation of students at school.

 Researchers identify that in the study of leadership in Indonesia there are many, but in the context of the effectiveness of leadership behavior there is still little. There is still no known successful and effective principal leadership behavior that can motivate teachers to improve performance. Thus, the researchers attempted to measure from 3 indicators, namely, first, how the relationship between leaders and followers was identified, such as the behavior of followers' beliefs towards the leader, behavior of followers' respect, behavior of leader intimacy, and behavior of followers' affection for the leader. Second, looking at effectiveness in terms of task structure through identification of clear goals, clarity of tasks to be carried out, level of diversity in problem solving encountered at work, level of openness regarding specific decisions faced. Third, the position of leaders and followers identified from the level of the leader's power in directing followers, the level of the leader's power, the strength of the leader in evaluating followers, the level of authority of the leader in rewarding followers, and the level of authority of the leader in punishing followers. If studied further, it will be seen that leadership behavior has an impact on student achievement and teacher performance. This is in line with Marlina (2013): Trust is a very significant thing in a management. Good management is supported by good leadership. Leadership stands on the foundation of trust. When confidence is fragile, then the leader will soon collapse (https://doi.org/10.19109/td.v18i02.47). Joni Bungai's research (2006) also revealed that there is a direct effect of teacher teaching effectiveness on the academic quality of graduates is 0.753 (http://dx.doi.org/10.17977/jip.v13i2.51).

 In Irwandy's research (2014): The objective of this study was to assess the role of motivation on teacher performance in Indonesia. This study is an assessment of this aim and used a deductive approach where a qualitative survey was conducted among students at the State University of Medan (UNIMED), Indonesia who are assumed to be future teachers. The survey was intended to get their responses on what they feel are the best factors that could motivate them as future teachers among a list of ten motivational factors. In the light of this, the study sets to identify the most ranked factors among the ten motivational factors. The analysis from the empirical findings showed that Good Salary was the most ranked factor for both sub-groups that made up the sample survey (DOI:10.7575/aiac.ijels.v.2n.2p.90). In Rudolf Kempa's research (2009), the results of the SEM analysis of the principal's managerial skills variable have a positive and significant relationship, significant on teacher performance. (http://dx.doi.org/10.17977/jip.v16i1.2542).

 Indonesia is a highly pluralistic country and diverse nation, reflected by its national credo: Bhinneka Tunggal Ika or Unity in Diversity. The mission of national education is conducted democratically, equally, and non- discriminatorily based on human rights, religious values, cultural values, and national pluralism. Educational streaming consists of formal education, nonformal education, and informal education that may complement and enrich each other (Act of the Indonesia, Number 20, Year 2003, Chapter VI, Article 13, Verse, 1) ( http://publikasi.data.kemdikbud.go.id/uploadDir/isi_B3FFDDBD-A1EF 4EF1-838F-7534337F7346_.pdf). School principals play an important role in improving education quality and accountability of education implementation in the educational unit. The problem commonly encountered by the principal is the weakness in managerial competence, while from the school supervisor is the lack of competence in supervisory skills. In the city of Tenggarong, the problem of the competence of the principal of public high schools cannot be explained specifically, but the student graduation rate is quite good. The determination of the number of graduates in 2014/2015 is no longer determined from the results of the National Examination (UN) but from the respective schools. The graduation rate of public high school students in Tenggarong decreased in the 2015/2016 academic year by 98.95% when compared to the previous year the number of high school graduates was 99.46% (source: school data). This is to the attention of researchers, that if academic achievement, namely the graduation rate is no longer determined nationally, then the effectiveness of principals' leadership and teacher performance needs to be reviewed and investigated.

 1. How are variables measured?

 The exogenous variable is the Effectiveness of Leadership Behavior Patterns, and two endogenous variables, namely Teacher Performance and School Achievement, are measured using a 5-level Likert scale where respondents are asked to state their attitude by marking a very high statement to a very low gradation. The effectiveness of the behavior pattern is measured through 3 manifest variables, namely the relationship between leader and followers (X〗_1), task structure (X〗_2), and strength of leader position (X_3). While the teacher's performance is seen from the manifest variables, namely: the level of teacher ability in formulating learning objectives in the lesson plans according to the curriculum; the level of the teacher's ability to plan learning activities effectively; the level of the teacher's ability to choose learning resources and learning media according to learning materials and strategies; the level of the teacher's ability to apply the approach and effective learning strategies; the level of the teacher's ability to encourage and maintain student involvement in learning; the level of the teacher's ability to end learning effectively; the level of the teacher's ability to design assessment methods and evaluation tools to measure the progress and success of students' learning. Student achievement is seen from school test scores and student report cards, then data is obtained and processed by descriptive statistical analysis. The results of measuring the effectiveness of the principal's leadership behavior, the average score (mean) obtained is 108.85. Looking at the standard deviation of the SPSS data processing or standard deviation of 22,959 with a median value of 122.00 and a value that occurs frequently (mode) of 132.23. The teacher performance variable has an average score (mean) of 115.26. Looking at the acquisition of the standard deviation or standard deviation of 19,975 with a median value of 122.00 and a value that occurs frequently (mode) of 133.80. The student achievement variable, the average score (mean) obtained for the US score is 80.74 and the report card score is 81.80. Looking at the standard deviation of the US value of 5,742 and 5,024 on the report card value, with a median value of 82.00 on the US value and 84.00 on the report card value, as well as the frequently occurring value (mode) of 86 on the US value, and 86.75 on the report card value. Furthermore, it is included in the evaluation of the proposed research model through the analysis of Goodness of Fit. After the initial model is processed with a sample of 281 respondents, the value of X^2 is generated along with the probability, the proposed research model can be accepted if there is a difference between the sample covariance matrix and the observed population covariance matrix. Next is the identification of the structural model that can be seen from the results of the count variable by calculating the amount of covariance and variance data compared to the number of parameters to be estimated. From these results, it produces a degree of freedom value and the sentence "minimum was archived", then the process of testing the maximum likelihood estimation has been carried out and the estimation is identified with the results of the data being normally distributed. Furthermore, the analysis of the relationships between constructs in the hypothesis is shown by the regression weight value.

 2. What does c.r mean?

 C.R is the critical ratio. Meets the conditions for data normality, namely -2.58 < c.r < 2.58. This means that all normal data are multivariate.

 3. What does *** mean?

 The meaning of *** = 0.00 (P-value is very small and is below 0.05).

 4. Indirect influence?

 To see the indirect effect of the principal's leadership variable on student achievement through teacher performance by comparing the values ​​of standardized direct effects and standardized indirect effects. This means that if the value of standardized direct effects is smaller than standardized indirect effects, it can be said that the mediating variable has an influence indirectly in the relationship between the two variables (independent and dependent).

 The implication of this research is that the relationship between leaders and followers in the behavioral effectiveness of leadership has the highest contribution value so that if this good relationship can continue, it is believed to be able to improve student achievement and teacher performance. However, in the results obtained, schools need to be concerned about teacher performance, 13.88% of respondents stated that it was quite low. This can be seen from the results of statistical measurements of teacher respondents' answers, the level of ability to encourage and maintain student involvement has the least influence and the level of ability to apply learning approaches and strategies. So if schools want to develop, they need to increase/focus on evaluating and developing learning approaches and strategies by teachers, as well as increasing student involvement in classroom learning.

# Peer review: Reviewer response for version 1

**DOI:** 10.5256/f1000research.54731.r87564

**Published:** 2021-07-09

**Authors:** Lokman Mohd Tahir

**Affiliations:** 1 School of Education, Universiti Teknologi Malaysia, Skudai, Malaysia

**Introduction**

1. When emphasising the vital role of principals, it would better to support this with previous research findings and citations to stress the importance of the sentence.
2. What is the research gap for this study? Please state the reason why this study was conducted.

**Literature review**

1. When stressing the importance of principals’ leadership, it is suggested to support with many citations.
2. The style of quoting the citation needs to be consistent.
3. There is a need to provide some literature on the previous principals’ leadership within the Indonesian schools.
4. I personally suggest to the authors to add many previous studies or past literature which have studied the effective principal leadership behaviors on teacher performance, effective principal leadership behaviors on student achievement, and teacher performance on student achievement.

**Methods, Results, and Discussion**

1. What is the significant reason to select only five schools?
2. Please proofread the article to delete any grammatical mistakes.
3. What are the implications of the study?
4. In reading the article, are there any similarities with previous studies which are presented in the literature review?

Is the work clearly and accurately presented and does it cite the current literature?

Partly

If applicable, is the statistical analysis and its interpretation appropriate?

Yes

Are all the source data underlying the results available to ensure full reproducibility?

Yes

Is the study design appropriate and is the work technically sound?

Yes

Are the conclusions drawn adequately supported by the results?

Yes

Are sufficient details of methods and analysis provided to allow replication by others?

Partly

Reviewer Expertise:

NA

I confirm that I have read this submission and believe that I have an appropriate level of expertise to confirm that it is of an acceptable scientific standard, however I have significant reservations, as outlined above.

# Response

**Published:** 2021-07-12

**Authors:** tria ina utari

**Affiliations:** IAIN AMBON, Indonesia

**Introduction**

 In the pre-observation conducted by researchers at schools, they found a tendency for principals who were less able to apply leadership acceptable to teachers. This slightly affected teacher performance in terms of: quality of work in classroom management; learning instruments were not developed enough; little initiative in working was seen when researchers, suddenly only a few, are in place; and the ability to communicate their work, as not all principals control the completeness of teachers in teaching. In the city of Tenggarong, the problem of the competence of the principal of public high schools cannot be explained specifically, but the student graduation rate is quite good. The determination of the number of graduates in 2014/2015 is no longer determined from the results of the National Examination (UN) but from the respective schools. The graduation rate of public high school students in Tenggarong decreased in the 2015/2016 academic year by 98.95%, when compared to the previous year the number of high school graduates was 99.46% (source: school data). This is to the attention of researchers, that if academic achievement, namely the graduation rate is no longer determined nationally, then the effectiveness of principals' leadership and teacher performance needs to be reviewed and investigated. According to Smith in Hendarman (2015), "to build an effective school it is necessary to have strong instructional leadership, clear attention to learning outcomes, high student rewards, a good environment and supervision at the level of achievement". Assuming the more effective one's leadership is, the higher the teacher's performance and school achievement will be.

**Literature review**

 As for this study about the effectiveness of the principal's leadership behavior pattern, teacher performance, and school achievement at SMA Negeri Tenggarong, the authors first found references and read some relevant sources conducted by the authors and other researchers as follows:

 According to Suyarno (2011) in his thesis about the relationship between the effectiveness of principals' leadership and teacher work motivation with the performance of senior high school teachers in Paser Regency, his research states that there is a significant relationship between the principal's leadership and performance.

 Marlina, Leny (2013): *Trust is a very significant thing in a management. Good management is supported by good leadership. Leadership stands on the foundation of trust. When confidence fragile, then the leader will soon collapse. Similar to a standing leadership in education on the basis of trust. Therefore, the most fundamental and important when being a leader is to instill confidence in the members or subordinates because of the way that a leader will be respected and honored in an organization. Planting a sense of trust in subordinates leadership is influenced by the type of person and the type of leadership style depends on the person.*

 Ratmawati (2011): *Principals’ Qualification, Training Intensity, Environment, Motivation, and Principals’ Competences. The study tries to investigate the relationship between the qualification, education-and-training intensity, environment, motivation, and competence of principals. The data collected using a questionnaire from 239 principals of Elementary Schools in Makassar are analyzed using multivariate analysis based on Structural Equation Models (SEM). The result shows that there is a significant correlation between the quali-fication, education-and-training intensity, environment, motivation, and competence of the principals*

 Irwandy (2013): *The objective of this study was to assess the role of motivation on teacher performance in Indonesia. This study is an assessment of this aim used deductive approach where a qualitative survey was conducted among students at Universitas Negeri Medan (UNIMED), Indonesia who are assumed to be future teachers. The survey was intended to get their responses on what they feel are the best factors that could motivate them as future teachers among a list of ten motivational factors. In the light of this the study sets to identify the most ranked factors among the ten motivational factors. The analysis from the empirical findings showed that Good Salary was the most ranked factor for both sub groups that made up the sample survey. However, a study from previous researches used in this study showed that different results could be obtained from different groups of already working employees. This study therefore can be seen as an introduction to a more detailed study to be carried by future researchers in the field of teachers’ motivation*

 Joni Bungai (2006) in his journal the influence of principal competence, school climate, and teaching effectiveness of teachers on the achievement of high school graduates in Central Kalimantan, in his research using SEM analysis stated that the direct influence of principal competence on the effectiveness of school infrastructure management in supporting learning activities teaching is 0.248, the direct influence of the principal's competence on the school climate is 0.122, the direct influence of the principal's competence on the effectiveness of teaching teachers is 0.084, the direct effect of school infrastructure on the effectiveness of teaching teachers is 0.072, the direct effect of school infrastructure on academic quality graduates is 0.131, the direct influence of school climate on the academic quality of graduates is 0.292, and the direct effect of teacher teaching effectiveness on the academic quality of graduates is 0.753. The indirect effect of the principal's competence on the effectiveness of teaching teachers is 0.087, the indirect effect of the principal's competence on the academic quality of graduates is 0.106, the indirect effect of school infrastructure management on the academic quality of graduates is 0.093 and the indirect effect of school climate on the academic quality of graduates is 0.032.

 Rudolf Kempa (2009) in his journal Leadership Behavior, managerial skills, conflict management, stress resistance and performance of State Elementary School teachers in Ambon City., in his research using SEM analysis stated that the leadership behavior variable has a positive and significant relationship to the managerial skills of school principals. . Thus, it can be said that the better leadership behavior will improve managerial skills. The results of the SEM analysis of the leadership behavior variable have a positive and significant relationship to conflict management. The results of the SEM analysis of the leadership behavior variable have a positive and significant relationship to the resilience of teacher work stress. The results of the SEM analysis of the managerial skills variable have a positive and significant relationship to conflict management. The results of the SEM analysis of the principal's managerial skills variable have a positive and significant relationship to the resilience of teacher work stress. The results of the SEM analysis of the principal's managerial skills variable have a positive and significant relationship. significant on teacher performance. The results of the SEM analysis of the conflict management variable have a negative and significant relationship to the resilience of teacher work stress. The results of the SEM analysis of the conflict management variable have a negative and significant relationship to teacher performance. The results of the SEM analysis of the teacher's work stress endurance variable have a positive and significant relationship to teacher performance. The results of the SEM analysis of the leadership behavior variable on conflict management through managerial skills have a positive and significant relationship. The results of the SEM analysis of the leadership behavior variable on the resilience of teacher work stress through managerial skills and conflict management have a positive and significant relationship. The results of the SEM analysis of the variables of leadership behavior on teacher performance through managerial skills, conflict management, and resilience of teacher work stress have a positive and significant relationship.

**Methods, Results, and Discussion**

1. The reason for choosing 5 schools is because this research is limited to only public high schools managed by the government, not including private schools, with the consideration that private schools already have and are the concern and development of local foundations. Then, after the high school population was limited to public schools, it was found that there were only 5 schools. So that the 5 school populations were selected as saturated samples, i.e. all schools were selected as samples because of the small population (Sugiyono, 2015).
2. The implication of the research shows that the effectiveness of the principal's leadership behavior pattern makes a very important contribution to improving the quality of teacher performance and school achievement. Effect of Effectiveness Patterns of Principal Leadership Behavior is the ability of principals to influence and direct teachers and all elements effectively and efficiently in order to achieve school goals together.
  The model for measuring the effectiveness of leadership behavior patterns can be used as an illustration that it is necessary to pay attention to the aspect of the leader's position, which has the smallest contribution. As for the level of leader power in directing followers and evaluating followers, the authority of the leader is in giving rewards to followers, giving punishment to followers, problem-solving abilities, level of openness, and diversity in solving problems.
  To achieve good and quality teacher performance, there needs to be considerable attention from the principal to the teachers, so that teachers still feel motivated and enthusiastic in carrying out their duties. The principal in his leadership is expected to improve the quality of teacher performance by heeding all attitudes, methods of guidance, supervision, and direction.
  Improving the quality of the principal's personality is needed and must be done considering that the principal is a figure in the school who truly becomes a role model for all school members.
3. That's right, the similarity of previous research is used as a reference in supporting the results of this study.

# Response

**Published:** 2021-07-23

**Authors:** tria ina utari

**Affiliations:** IAIN AMBON, Indonesia

**Introduction**

 In the pre-observation conducted by researchers at schools, they found a tendency for principals who were less able to apply leadership acceptable to teachers. This slightly affected teacher performance in terms of: quality of work in classroom management; learning instruments were not developed enough; little initiative in working was seen when researchers, suddenly only a few, are in place; and the ability to communicate their work, as not all principals control the completeness of teachers in teaching. In the city of Tenggarong, the problem of the competence of the principal of public high schools cannot be explained specifically, but the student graduation rate is quite good. The determination of the number of graduates in 2014/2015 is no longer determined from the results of the National Examination (UN) but from the respective schools. The graduation rate of public high school students in Tenggarong decreased in the 2015/2016 academic year by 98.95%, when compared to the previous year the number of high school graduates was 99.46% (source: school data). This is to the attention of researchers, that if academic achievement, namely the graduation rate is no longer determined nationally, then the effectiveness of principals' leadership and teacher performance needs to be reviewed and investigated. According to Smith in Hendarman (2015), "to build an effective school it is necessary to have strong instructional leadership, clear attention to learning outcomes, high student rewards, a good environment and supervision at the level of achievement". Assuming the more effective one's leadership is, the higher the teacher's performance and school achievement will be.

**Literature review**

 As for this study about the effectiveness of the principal's leadership behavior pattern, teacher performance, and school achievement at SMA Negeri Tenggarong, the authors first found references and read some relevant sources conducted by the authors and other researchers as follows:

 According to Kailola, L.G  (2017) in his thesis about the principal leadership and work motivation with teacher performance at smk negeri putussibau-kapuas hulu, his research states that there is a significant relationship between the principal's leadership and teacher performance, There is a positive and significant relationship between work motivation and teacher performance, there is a positive and significant relationship between principal leadership and work motivation and teacher performance. Retrieved from http://ejournal.uki.ac.id/index.php/jmp/article/view/332

 Marlina, Leny (2013): *Trust is a very significant thing in a management. Good management is supported by good leadership. Leadership stands on the foundation of trust. When confidence fragile, then the leader will soon collapse. Similar to a standing leadership in education on the basis of trust. Therefore, the most fundamental and important when being a leader is to instill confidence in the members or subordinates because of the way that a leader will be respected and honored in an organization. Planting a sense of trust in subordinates leadership is influenced by the type of person and the type of leadership style depends on the person.* https://doi.org/10.19109/td.v18i02.47

 Ratmawati (2011): *Principals’ Qualification, Training Intensity, Environment, Motivation, and Principals’ Competences. The study tries to investigate the relationship between the qualification, education-and-training intensity, environment, motivation, and competence of principals. The data collected using a questionnaire from 239 principals of Elementary Schools in Makassar are analyzed using multivariate analysis based on Structural Equation Models (SEM). The result shows that there is a significant correlation between the quali-fication, education-and-training intensity, environment, motivation, and competence of the principals. *DOI: 10.17977/jip.v17i6.2880

 Irwandy (2014): *The objective of this study was to assess the role of motivation on teacher performance in Indonesia. This study is an assessment of this aim used a deductive approach where a qualitative survey was conducted among students at Universitas Negeri Medan (UNIMED), Indonesia who are assumed to be future teachers. The survey was intended to get their responses on what they feel are the best factors that could motivate them as future teachers among a list of ten motivational factors. In the light of this the study sets to identify the most ranked factors among the ten motivational factors. The analysis from the empirical findings showed that Good Salary was the most ranked factor for both sub-groups that made up the sample survey. However, a study from previous researches used in this study showed that different results could be obtained from different groups of already working employees. This study therefore can be seen as an introduction to a more detailed study to be carried by future researchers in the field of teachers’ motivation. *DOI: https://dx.doi.org/10.7575/aiac.ijels.v.2n.2p.90 

 Joni Bungai (2006) in his journal the influence of principal competence, school climate, and teaching effectiveness of teachers on the achievement of high school graduates in Central Kalimantan, in his research using SEM analysis, stated that the direct influence of principal competence on the effectiveness of school infrastructure management in supporting learning activities teaching is 0.248, the direct influence of the principal's competence on the school climate is 0.122, the direct influence of the principal's competence on the effectiveness of teaching teachers is 0.084, the direct effect of school infrastructure on the effectiveness of teaching teachers is 0.072, the direct effect of school infrastructure on academic quality graduates is 0.131, the direct influence of school climate on the academic quality of graduates is 0.292, and the direct effect of teacher teaching effectiveness on the academic quality of graduates is 0.753. The indirect effect of the principal's competence on the effectiveness of teaching teachers is 0.087, the indirect effect of the principal's competence on the academic quality of graduates is 0.106, the indirect effect of school infrastructure management on the academic quality of graduates is 0.093 and the indirect effect of school climate on the academic quality of graduates is 0.032. DOI: http://dx.doi.org/10.17977/jip.v13i2.51

 Rudolf Kempa (2009) in his journal Leadership Behavior, managerial skills, conflict management, stress resistance and performance of State Elementary School teachers in Ambon City., in his research using SEM analysis stated that the leadership behavior variable has a positive and significant relationship to the managerial skills of school principals. . Thus, it can be said that the better leadership behavior will improve managerial skills. The results of the SEM analysis of the leadership behavior variable have a positive and significant relationship to conflict management. The results of the SEM analysis of the leadership behavior variable have a positive and significant relationship to the resilience of teacher work stress. The results of the SEM analysis of the managerial skills variable have a positive and significant relationship to conflict management. The results of the SEM analysis of the principal's managerial skills variable have a positive and significant relationship to the resilience of teacher work stress. The results of the SEM analysis of the principal's managerial skills variable have a positive and significant relationship. significant on teacher performance. The results of the SEM analysis of the conflict management variable have a negative and significant relationship to the resilience of teacher work stress. The results of the SEM analysis of the conflict management variable have a negative and significant relationship to teacher performance. The results of the SEM analysis of the teacher's work stress endurance variable have a positive and significant relationship to teacher performance. The results of the SEM analysis of the leadership behavior variable on conflict management through managerial skills have a positive and significant relationship. The results of the SEM analysis of the leadership behavior variable on the resilience of teacher work stress through managerial skills and conflict management have a positive and significant relationship. The results of the SEM analysis of the variables of leadership behavior on teacher performance through managerial skills, conflict management, and resilience of teacher work stress have a positive and significant relationship. DOI: http://dx.doi.org/10.17977/jip.v16i1.2542

**Methods, Results, and Discussion**

1. The reason for choosing 5 schools is because this research is limited to only public high schools managed by the government, not including private schools, with the consideration that private schools already have and are the concern and development of local foundations. Then, after the high school population was limited to public schools, it was found that there were only 5 schools. So that the 5 school populations were selected as saturated samples, i.e. all schools were selected as samples because of the small population (Sugiyono, 2015)
2. The implication of the research shows that the effectiveness of the principal's leadership behavior pattern makes a very important contribution to improving the quality of teacher performance and school achievement. Effect of Effectiveness Patterns of Principal Leadership Behavior is the ability of principals to influence and direct teachers and all elements effectively and efficiently in order to achieve school goals together.
  The model for measuring the effectiveness of leadership behavior patterns can be used as an illustration that it is necessary to pay attention to the aspect of the leader's position, which has the smallest contribution. As for the level of leader power in directing followers and evaluating followers, the authority of the leader is in giving rewards to followers, giving punishment to followers, problem-solving abilities, level of openness, and diversity in solving problems.
  To achieve good and quality teacher performance, there needs to be considerable attention from the principal to the teachers, so that teachers still feel motivated and enthusiastic in carrying out their duties. The principal in his leadership is expected to improve the quality of teacher performance by heeding all attitudes, methods of guidance, supervision, and direction.
  Improving the quality of the principal's personality is needed and must be done considering that the principal is a figure in the school who truly becomes a role model for all school members.
3. That's right, Prof, the similarity of previous research is used as a reference in supporting the results of this study.
